# Seeing Double: A Case of ACL Tears in Monozygotic Twin Female Athletes Within 48 Hours

**DOI:** 10.7759/cureus.7244

**Published:** 2020-03-11

**Authors:** Jessica N Pelkowski, Kristina DeMatas, Ryan C McCoy, Cedric J Ortiguera

**Affiliations:** 1 Orthopedic Surgery, Mayo Clinic, Jacksonville, USA; 2 Family Medicine/Sports Medicine, Mayo Clinic, Jacksonville, USA; 3 Physical Therapy, Mayo Clinic, Jacksonville, USA

**Keywords:** twins, acl tear, sports injury, twin study, anterior cruciate ligament (acl)

## Abstract

Anterior cruciate ligament (ACL) injuries are a serious issue for young athletes. These injuries are devastating and costly, leading to significant time away from sports, and substantial financial cost. A case is presented in which monozygotic twin sisters sustained ACL injuries within 48 hours of one another. Limited research is available studying twins with ACL tears and the risk factors associated with them. This is the first reported case of monozygotic sisters that are high-level athletes with ACL tears in such close proximity to one another. Enhanced knowledge of genetic contribution to ACL injuries is important to better understand predisposing factors and to develop preventative approaches. More research is needed to support a distinct association between ACL rupture and genetic variants.

## Introduction

Anterior cruciate ligament (ACL) injuries are a serious issue for young athletes with over 100,000 incidences annually in the United States [1]. The highest incidence occurs in female athletes aged 14-18 [2-3]. A multitude of both modifiable and nonmodifiable risk factors have been reported in the literature regarding ACL tears, including female gender, increased valgus joint loading, increased ligamentous laxity, decreased intercondylar notch width, and decreased core and hip strength [1, 4-5]. Females exhibit kinematic differences compared with males, including a decreased ability to reduce force when landing, altered quadriceps and hamstring activation, and increased dynamic knee abduction [4]. A family history of ACL tear may increase the risk of an ACL tear likely because of heritable factors, such as biomechanical, anatomic, anthropometric, and neuromuscular traits [4]. Hormonal cyclic variations have been proposed to affect the laxity of ligaments and possibly the incidence of ACL injury [4]. Familial predisposition and gene variants may also be significant risk factors for non-contact ACL injuries [5].

Limited research is available studying twins with ACL tears and the risk factors associated with them [6-7]. Enhanced knowledge of genetic contribution to ACL injuries is important to better understand predisposing factors and to develop preventative approaches [8]. More research is needed to support a distinct association between ACL rupture and genetic variants. 

An ACL injury is a devastating and costly condition leading to significant time away from sports and substantial financial cost. A better understanding of the modifiable risk factors is essential for ACL injury prevention and may help mitigate the non-modifiable risk factors. This is the first reported case of monozygotic sisters that are high-level athletes with ACL tears within 48 hours.

## Case presentation

**Figure 1:**
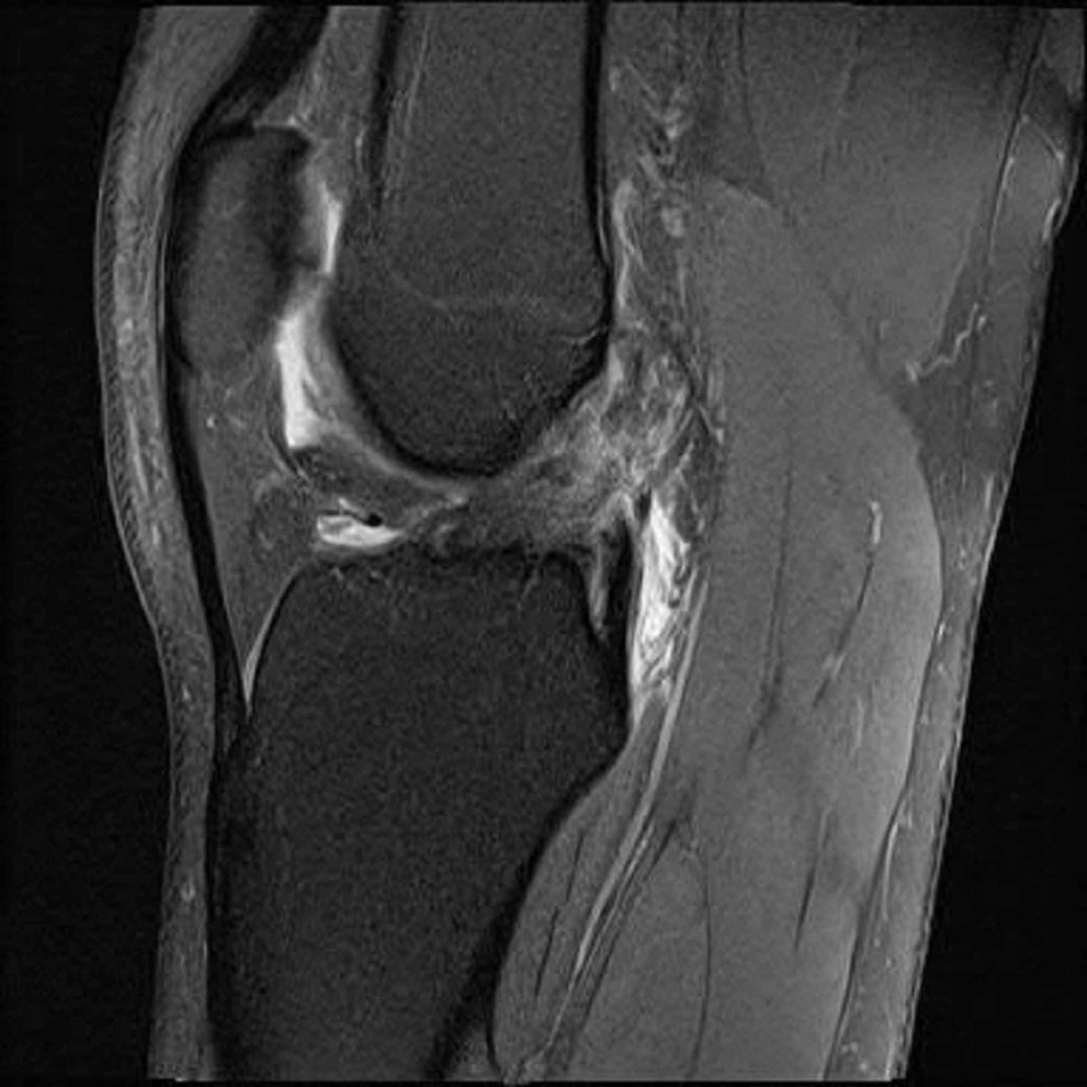
Magnetic resonance imaging (MRI) confirming an anterior cruciate ligament (ACL) tear with associated bone contusions in Patient A

A 17-year-old Caucasian female (Patient A) presented to the outpatient sports medicine clinic for evaluation of acute onset left knee pain and swelling. Earlier that day, she was at basketball practice for her high school team when she landed on her left knee following a jump. At the time of landing, she experienced a pop that was accompanied by knee-buckling and a fall. She experienced immediate medial knee pain with tightness in her posterior knee. Her physical examination was remarkable for a small knee effusion, active range of motion 0 to 90 degrees, positive Lachman test, a positive valgus stress test for pain but no gapping was present, and equivocal McMurray’s test. Magnetic resonance imaging (MRI) was obtained and confirmed a complete ACL tear with associated bone contusions and partial grade II medial collateral ligament (MCL) sprain (Figure 1). She began physical therapy five days later for swelling and range of motion. 

**Figure 2:**
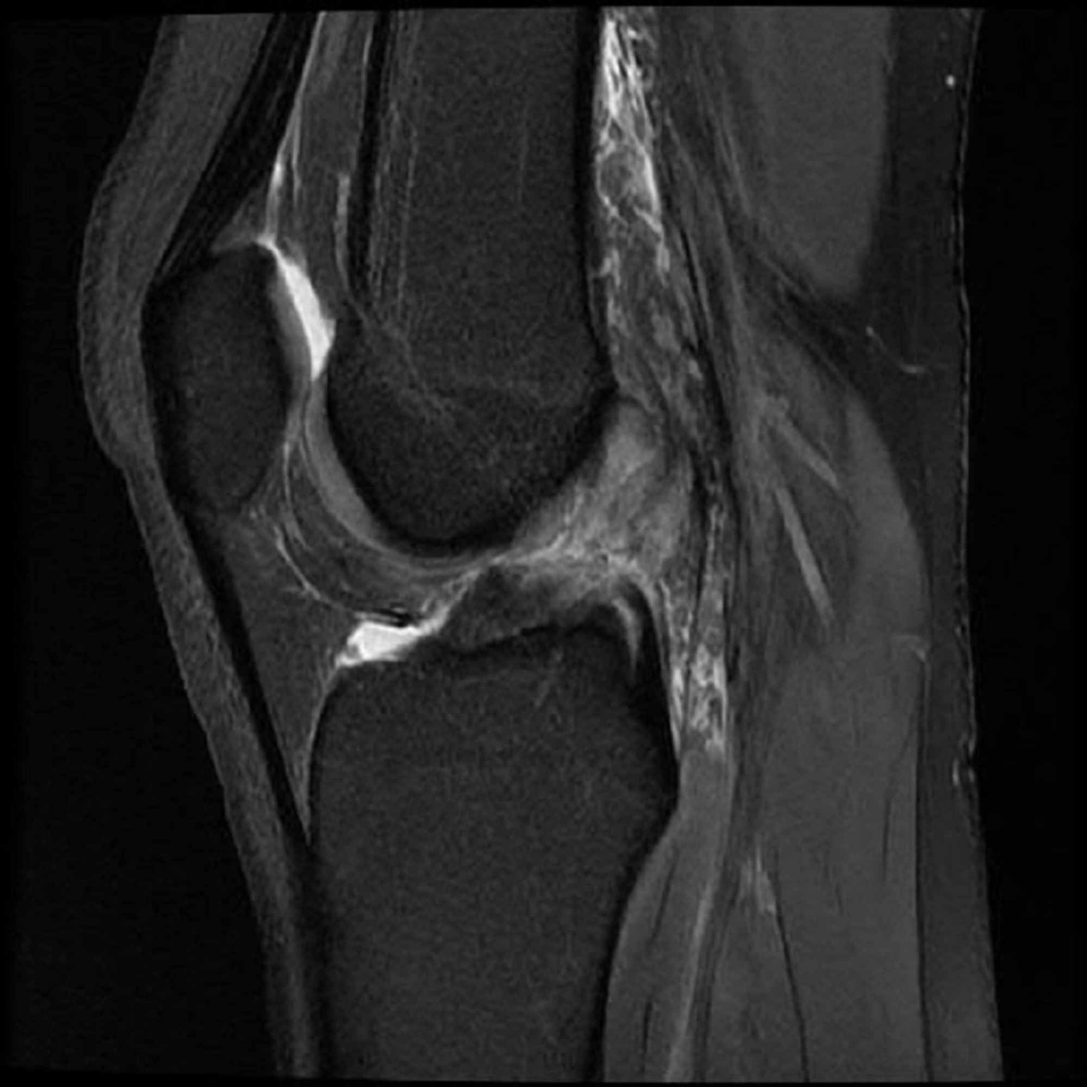
Magnetic resonance imaging (MRI) which showed a complete anterior cruciate ligament (ACL) tear with associated bone contusions in Patient B

Two days later, her identical twin sister (Patient B), who plays on the same basketball team, was playing in a basketball game when she sustained a twisting injury to her left knee with acute onset of pain. She presented to the sports medicine clinic the same day with a report of instability and swelling. She was ambulating with minimal difficulty and minimal pain. Her physical examination was remarkable for a small knee effusion, normal active range of motion, positive Lachman test, negative valgus stress test, and negative McMurray’s test. An MRI scan was obtained and showed a complete ACL tear with associated bone contusions (Figure 2). No meniscal tears were present. She began physical therapy a week later. 

Both patients were in the ovulatory menstrual phase at the time of their ACL injuries and Patient A’s last menstrual period was five days before Patient B’s. Neither patient was on oral contraceptive pills (OCP) at the time of injury. The onset of menarche was at the age of 13 and both patients’ menstrual cycles were regular.

Because of the patients’ young ages and desires to remain active, ACL reconstruction was recommended. The options of patellar tendon versus hamstring autograft were presented to them and their parents at the consultations with the same orthopedic surgeon. They both elected to proceed with hamstring autograft. Patient A underwent left knee arthroscopy with ACL reconstruction using a hamstring autograft three weeks and six days post-injury. Patient B underwent left knee arthroscopy with ACL reconstruction using a hamstring autograft two weeks and five days post-injury. Although both twins sustained similar tears in their ACLs, Patient A struggled more with obtaining a full range of motion and swelling control following the injury. It was for this reason that her surgery was after her sister’s despite her injury occurring two days earlier. 

Both patients began pre-rehabilitation within one week of injury to improve outcomes following ACL reconstruction [9]. Patient A presented to therapy with increased subjective pain levels, joint line swelling, knee range of motion lacking five degrees of extension to 95 degrees of flexion, 3+/5 left lower extremity hip abduction manual muscle test (MMT), 5/5 upper abdominal and 4/5 lower abdominal testing, and good proprioception of the lower extremity with closed kinetic chain activities. Patient B presented to therapy with no reported pain, no joint line swelling, knee range of motion lacking seven degrees of extension to 134 degrees of flexion, 3+/5 hip abduction left lower extremity MMT, 5/5 upper abdominal and 3+/5 lower abdominal testing, and required moderate verbal and tactile cueing to increase spine and lower extremity proprioception during closed kinetic chain activities. Both patients finished therapy with pain-free, normal knee range of motion, no swelling, excellent activation of quadriceps, repetitive straight leg raises without an extensor lag, and the ability to perform pain-free repetitive squats without any corrective cueing. Due to anticipated surgical intervention, quadriceps isokinetic testing was not performed to reduce the risk of effusion.

Both patients started postoperative physical therapy within one week of their respective surgeries with the same physical therapist as pre-rehabilitation. The identical physician protocol was used for both patients. Both patients achieved expected milestones through six weeks [10]. Nearly seven weeks postoperatively, Patient B experienced swelling to her knee with increased pain. There was no trauma or identifiable cause of these symptoms. Her physical therapy transitioned to focusing on reducing pain and swelling and preventing inhibition of her quadriceps. She was delayed by four to five weeks for milestone achievement. 

Both patients progressed well and achieved all milestones. At 9.5 weeks postoperatively, Patient A began running progression. At 12 weeks postoperatively, she was running one mile three times weekly, performing footwork and basketball handling drills, and shooting free throws. At 12 weeks postoperatively, Patient B began running progression, as well as the same supervised exercise progression as Patient A. Both patients have been cleared for full activity and have returned to basketball.

## Discussion

In this interesting and unfortunate case, monozygotic twin sisters sustained ACL injuries within 48 hours of one another. Current injury prevention programs strive to identify modifiable risk factors associated with an ACL injury. A recent review showed a significant reduction in injury rate when athletes received injury prevention programs [7]. The National Athletic Trainers Association also supports multicomponent injury-prevention training programs to reduce ACL and knee injuries [11]. Both patients play on the same basketball team with the same training regime and participated in the same off-season training. No specific ACL preventative program was in place prior to injuries despite evidence that neuromuscular training in adolescent athletes increases dynamic knee stability, thus reducing ACL injury risk [7]. Both patients participated in a traditional team-based strength and conditioning program in the training facility at their school. This included but was not limited to free weights, machine-based strengthening, core programs, and stretching/cardio drills on the basketball court. 

There are several studies and case reports that suggest associations between knee disorders or injuries and various genetic variants, possibly suggesting that genetic predisposition is an important factor in knee disorders or injuries. Genetic associations are not limited to ACL injuries and some studies investigating similar knee conditions in twins involve bony conditions. Richie and Sytsma reported a case of matching osteochondritis dissecans (OCD) lesions in monozygotic twin brothers and suggested the genetic component could lead to early identification and treatment of these lesions [13]. Similarly, Gans et al reported multiple case reports of twins with OCD lesions and that there may be a genetic component to its etiology [14]. Beamish, Roberts, and Cnuddle reported a case of monozygotic brothers that were high-level gymnasts with near-identical avulsion fractures of the inferior pole of the patella [12]. Although their injuries were 14 months apart, they were aged 13 and 15 at the time of their injuries, similar to the ages of patients in the other mentioned cases. These cases focusing on bony injuries all suggest a genetic influence on bone mineral density and susceptibility to fracture [12-14].

As ACL tears have significant physical, mental, emotional, and economical consequences, there have been numerous cases, studies, and reviews examining both extrinsic and intrinsic risk factors for ACL tears. Extrinsic factors include the type of sport, level of activity, footwear and gear used, and playing surface [5, 15]. Intrinsic risk factors include age, gender, neuromuscular factors, cognitive factors, genetic factors, and hormonal factors. Anatomical risk factors include ACL size, body mass index, foot pronation, lower extremity joint laxity, decreased hamstring to quadriceps (H/Q) pelvic tilt, femoral intercondylar notch width, Q angle, and tibial slope [5, 15]. With respect to these intrinsic factors, there are some cases, studies, and reviews examining genetic variants in ACL injuries. However, this is the first reported case of monozygotic sisters that are high-level athletes with ACL tears within 48 hours. 

Magnussen et al. used the world’s largest twin registry, the Swedish Twin Registry, to report that ACL injury in both twins occurred more frequently in monozygotic (7.8%) than in dizygotic (1.5%) twins [8]. Multiple studies and case reports have reported that family history in a first-degree relative as a risk factor for an ACL tear. Data quantifying this risk is conflicting, with a range of 4% - 35% of patients with ACL tear having a family history of ACL tear [4, 16]. Flynn et al. reported an even higher statistic: that patients with ACL injuries were twice as likely to have a first-degree relative with a history of ACL injury when compared to a control group without a history of ACL injury [17]. A family history of ACL tear may increase the risk of ACL tear likely because of heritable factors such as biomechanical, anatomic, anthropometric, and neuromuscular traits. One trait hypothesized by Keays, Keays, and Newcombe is that siblings with narrow intercondylar notches are at increased risk of ACL tears as this is a known risk factor for an ACL tear and siblings would likely share this trait [1]. 

Sonn and Caltoum reported a case of 15-year-old monozygotic brothers that were high-level athletes with congenital absence of the right ACL that subsequently both underwent ACL construction with bone-patellar tendon-bone autograft [18]. Astur et al. reported a case of twin brothers who were both professional judo athletes that sustained similar ACL tears one year apart but underwent surgery on the same day by the same surgeon that utilized the same technique and graft type (hamstring tendon graft with femoral and tibial interference screws) [6]. Both patients were Tanner stage III at the time of their ACL tears. This case allowed for the comparison of two patients with a similar skeleton, age, height, weight, and genetic features. Caso et al. performed whole gene exome sequencing on twin brothers that both sustained ACL injuries, as well as their parents [19]. It was not noted if these twins were monozygotic or dizygotic. Eleven variants shared by family members were found to be associated with a non-contact ACL injury. However, the variants studied account for only a small number of the genes involved in the biology of the ACL [15, 19]. On the contrary, a systematic review by Kaynak et al. found conflicting evidence for some of the genetic variants identified by Caso et al. and evidence was insufficient to conclude if and which genetic variants had a role in ACL rupture [15]. Some potential genetic variants could influence the risk of an ACL injury but more data with large samples and phenotype homogeneity is needed to support an association between ACL injury and genetic variants.

There is also evidence that the menstrual cycle may affect anterior knee laxity and the risk of ACL rupture. It is estimated that women have a two to four-fold increased risk of ACL tear compared to men; however, how much of this risk is related to hormonal fluctuations remains unclear [20]. The direct associations between the menstrual cycle and ACL injury remain controversial. A recent systematic review with meta-analysis concluded an increased risk of ACL tear does not appear to be associated with periods of increased laxity. The results showed anterior knee translation was the lowest during the follicular phase (days 1-9) compared to the ovulatory phase (days 10-14) or the luteal phase (days 15-28) [20]. During the ovulatory phase, the surge in estradiol has been shown to decrease fibroblast proliferation and collagen synthesis, thus increasing knee laxity. Both patients were in the ovulatory phase of their menstrual cycle which may have caused increased laxity of the ACL, predisposing them to injury. Most studies regarding hormonal factors and ACL injuries are heterogeneous, have methodological differences, and are difficult to standardize due to the variability of the menstrual cycle. Additional higher-quality studies are certainly needed. A recent systematic review found there is limited evidence to support the association between OCP use and a reduction of ACL injuries [3]. These studies had small sample sizes and several methodological concerns; therefore, further studies are needed to evaluate the true association.

To conclude, these monozygotic twin athlete sisters likely had multiple variables that predisposed them to an ACL injury. We theorize that the combination of improper neuromuscular control, genetic factors, and possibly hormonal factors contributed to their ACL injuries. Both patients passed all standard ACL return to sports protocol testing and returned to sports six months postoperatively. Both continue to progress well with the goal of playing high-level college basketball. 

## Conclusions

A family history of ACL tear may increase the risk of an ACL tear. This is likely because of heritable factors, such as biomechanical, anatomic, anthropometric, and neuromuscular traits. Although a genetic predisposition to ACL injury is likely, the level of evidence remains low due to the rarity of cases and low sample size. Additional studies are recommended to examine genetic variants as a risk factor for ACL tears and other knee disorders. Comparisons of siblings, including twins, may provide clarity regarding genetic traits and predisposition to disorders or injuries. Enhanced knowledge of genetic contribution to ACL injuries is important to better understand predisposing factors and to develop preventative approaches. More research is needed to develop a standard ACL prevention protocol and to support a distinct association between ACL rupture and genetic variants. Limited research is available studying twins with ACL ruptures and the risk factors associated with them.
